# Service design oriented multidisciplinary collaborative team care service model development for resolving drug related problems

**DOI:** 10.1371/journal.pone.0201705

**Published:** 2018-09-28

**Authors:** Nayoung Han, Seung Hee Han, Hyuneun Chu, Jaehyun Kim, Ki Yon Rhew, Jeong-Hyun Yoon, Nam Kyung Je, Sandy Jeong Rhie, Eunhee Ji, Euni Lee, Yon Su Kim, Jung Mi Oh, for the DrugTEAM Study group

**Affiliations:** 1 College of Pharmacy and Research Institute of Pharmaceutical Sciences, Seoul National University, Seoul, Republic of Korea; 2 College of Pharmacy, Dongduk Women's University, Seoul, Republic of Korea; 3 College of Pharmacy, Pusan National University, Busan, Republic of Korea; 4 Division of Life and Pharmaceutical Sciences Graduate School, Ewha Womans University, Seoul, Republic of Korea; 5 College of Pharmacy, Gachon University, Incheon, Republic of Korea; 6 Kidney Research Institute, Seoul National University College of Medicine, Seoul, Republic of Korea; University of Hong Kong, HONG KONG

## Abstract

Our goal was to help prevent drug-related morbidity and mortality by developing a collaborative multidisciplinary team care (MTC) service model using a service design framework that addressed the unmet needs and perspectives of diverse stakeholders. Our service model was based on a “4D” framework that included Discover, Define, Design, and Develop phases. In the “discover” phase, we conducted desk research and field research of stakeholders to identify the unmet needs in existing patient care services. We used service design tools, including service safaris, user shadowing, and customer journey maps to identify pain and opportunity points in the current services. We also performed focus group discussions and in-depth interviews with stakeholders to explore the needs for improved services. In the “define” phase, we generated the service concept by mind mapping and brainstorming about the needs of stakeholders. The service concept was defined to be a Patient-oriented, Collaborative, Advanced, Renovated, and Excellent (P-CARE) service. We named the service “DrugTEAM” (Drug Therapy Evaluation And Management). In the “design” phase, we designed and refined four prototypes based on results from validation tests for their application towards following services: 1) medication reconciliation, 2) medication evaluation and management, 3) evidence-based drug information, and 4) pharmaceutical care transition services. During the “develop” phase, we implemented four services in a longitudinal chronic care model, considering the time spent by patients for each inpatient and outpatient setting. In conclusion, this is a study to develop a collaborative MTC service model using service design framework, focused on managing the unmet needs of patients and healthcare providers. As a result of implementing this service model, we expect to strengthen the professional relationship between pharmacists and stakeholders to ultimately create better patient outcomes.

## Introduction

Patients who take multiple medications due to chronic disease have a high risk of drug duplication, interaction, or adverse side effects, which could result in extended hospital stays and higher costs [1]. To increase the safety and effectiveness of treatment, these patients must have specific needs met, with regards to appropriate medication use [2]. In this regard, the pharmacist plays a key role in improving treatment outcomes by reconciling and monitoring medication use.

Recently, there has been a growing trend towards involving pharmacists in a multidisciplinary team of professionals that cooperates to comprehensive manage the demand for services [3]. Previous studies have shown that pharmacists’ services had positive effects on clinical and economic outcomes [4–6]. These pharmacist services included participation in rounds to optimize medication use, therapeutic drug monitoring, and providing drug information, patient and caregiver education, or medication reconciliation at admission or discharge. In addition, several studies have reported that multidisciplinary team care (MTC) service improved patient outcomes compared to user-cared patients [7, 8]. MTC’s goal is to provide high-quality treatment and improve the quality of care for patients in cooperation with healthcare professionals [9]. However, in South Korea, the development of MTC service is in the beginning stages, and healthcare professionals still independently provide services to patients. Furthermore, services in hospital pharmacies are limited to medication counseling, anticoagulation counseling, therapeutic drug monitoring, and similar services, despite the high risk of medication-related problems [10]. For this reason, advanced pharmaceutical care services for patients who may have medication-related problems are in high demand. Additionally, a team approach, such as MTC, is necessary to properly care for patients with chronic complex diseases.

A design approach for creating new services by incorporating the tangible and intangible elements of service was introduced to meet the needs of stakeholders [11, 12]. Tangible elements of a service include the hospital facility, computers, automobiles, and medicines; intangible elements include service plan, customer needs, and service delivery performance [11]. This service design approach starts by identifying the needs or expectations of new services, which are based on a wide range of experiences, to the current service provided by various stakeholders [13]. When this approach is applied to patient care, it is possible to develop new services that satisfy the unmet needs of stakeholders who affect or are affected by the service, such as patients, physicians, and nurses. Because pharmacists can interact with other healthcare providers as part of a multidisciplinary team, they play an important role in improving the understanding the thoughts and needs of stakeholders. Therefore, the aim of this study is to apply service design methodology to develop an advanced and collaborative MTC service model to address medication use, particularly for chronic diseases patients.

## Methods

**Fig 1:**
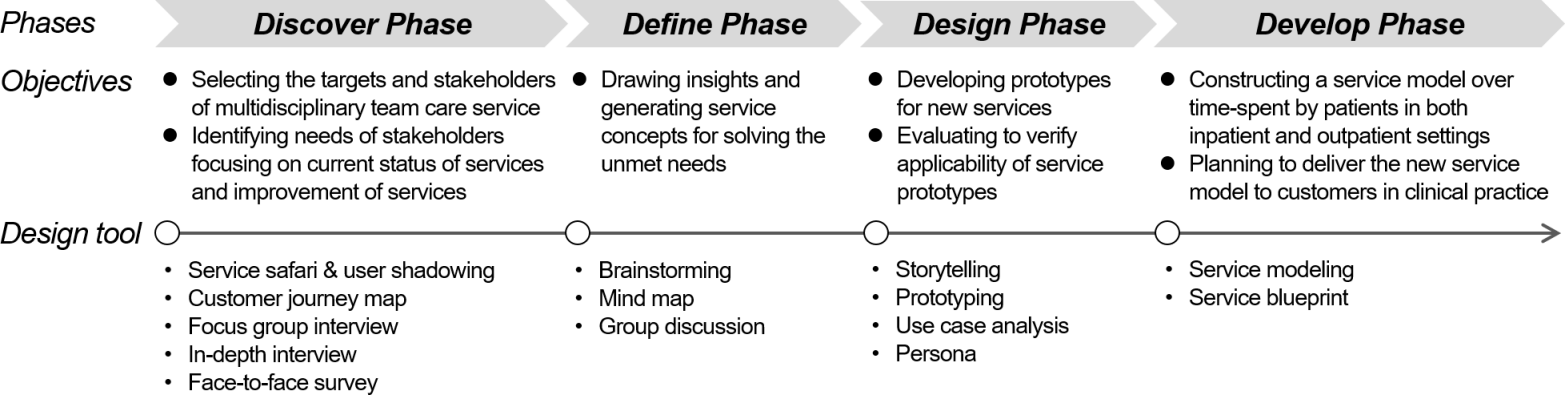
Service design framework for developing a collaborative multidisciplinary team care service model.

As shown in Fig 1, the service model development process followed a “4D” framework that included four phases: discover, define, design, and develop. For each phase, the respective objectives were to discover unmet needs, to define service concepts that would create insight, to design a set of services that would solve identified needs, and to develop an employable service model.

### The “discover” phase

**Fig 2:**
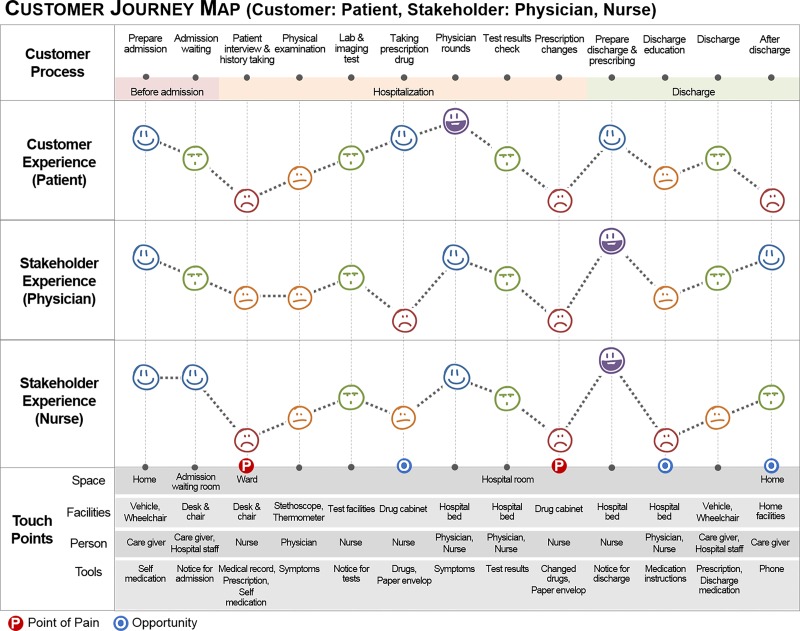
The customer journey maps of a patient, a physician, and a nurse.

The discover phase consisted of three steps, which attempted to identify the diverse needs and expectations of stakeholders. First, we selected service target groups and MTC service stakeholders by considering the priority of service requirements and using an analytic hierarchy process (AHP) survey with previously described methods [14]. In the second step, we explored the current perceptions of MTC services and identified unmet needs of various stakeholders, while focusing on service improvement. We gathered various stakeholders with an interest in collaborative MTC services, such as physicians, nurses, patients, and pharmacists. “Service safaris” [15] and “user shadowing” [16] were conducted to understand how the group felt about the currently available services and the sources of their problems during hospital stays. Service safaris and user shadowing are research methods to help understand how stakeholders interact with the service [15, 16]. We shadowed stakeholders and recorded their facial expressions and behaviors during activities related to patient care. Then, we represented and visualized how their emotional responses using a 1 to 5 interval scale as follows: “Not at all Satisfied,” “Partly Satisfied,” “Satisfied,” “More than Satisfied,” and “Very Satisfied”. We defined a “pain point” as a point where all three stakeholders were not at all satisfied, and defined an “opportunity point” as when one of the three stakeholders was not at all satisfied. The “customer journey maps” allowed us to see which parts of the service might need improvement (Fig 2) [16]. Finally, we conducted focus group discussions and in-depth interviews with 18 physicians and 15 nurses to gather information about their interests and discomfort in current services, as well as their expectations for new MTC services [10]. We also completed face-to-face surveys with 219 patients and focused on identifying their needs for improved MTC services [10, 17]. The resulting interview data was coded into themes and categories of decision-making processes [18, 19]. The codes were then interpreted by comparing the frequencies, co-occurrences, and relationships between the different themes [20]. All field research protocol for the identification of needs was approved by Institutional Review Board of Seoul National University (IRB No. 1401/001-013).

### The “define” phase

We interpreted and translated all findings from the desk and field research to generate a collaborative MTC service concept. We explored creative solutions for pain and opportunity points by group discussion and brainstorming. We generated ideas through the visual “mind mapping” process and grouped the ideas into clusters to identify meaningful insights. As a result of clustering, we selected the concept of our new service and established the objective of creating a collaborative MTC service model.

### The “design” phase

The design phase consisted of two steps: first designing and then validating prototypes. Using the service insights that were generated by mind mapping, we designed new services that would manage the unmet stakeholder needs. We selected service tasks from existing service guidelines, such as the core clinical pharmacy services that were proposed by the American Society of Hospital Pharmacists [21, 22], or clinical guidelines for target chronic diseases [23–25]. The resulting designed service was created as a prototype that included several components, including service objectives, service target, service providers, service content or tasks, service procedure at the time of the visit, and application methods and fees. The prototype was presented as a story or a series of short stories, by applying a storytelling method that contextualized the who, what, when, where, why, and how (5W1H) of each service.

We evaluated prototypes to verify that they could be applied to several conflicting situations, using a “use cases analysis” method. The analysis was conducted to simulate a stakeholder interaction with the developed services [15]. We conducted the methods by generating a fictional patient profile from existing patients. By using the created personas and use case analysis, we identified problems and stakeholders’ perspectives during application of the new services by observing the interaction between the stakeholders and the service prototypes in several situations. We repeated this feedback process to refine the interaction and ensure that services would resolve unmet needs.

### The “develop” phase

Based on the service prototypes, we developed a final service model for the time that patients spent in the hospital including both the circumstances of inpatient and outpatient settings. Because the collaborative MTC service model was intended to help provide continuous and longitudinal services for chronic disease patients, we based the model on a cyclical framework. We demonstrated the final service model using service blueprints from the points of view of both healthcare providers and patients.

## Results

### The “discover” phase

Using AHP analysis, we determined that the priority groups that would require collaborative MTC services were patients with diabetes mellitus, chronic heart disease, and chronic kidney disease [14]. Through qualitative, quantitative, and service design research, we found that physicians and nurses regularly were unable to obtain accurate patient medication histories, patient compliance, and information about new drugs (Fig 2) [10]. From the patient perspective, patients were frequently burdened by medication self-management, which is a problem exacerbated by common co-morbidities and/or the use of multiple medications [17]. We also found that patients wanted to receive special services at all time-points, including before and after meeting with a physician, after receiving a prescription, and after dispensing medication at a hospital [17]. Thus, the unmet needs were identified as: unintended medication discrepancies during hospital admission, inappropriate medication use in complex treatment regimens, low medication compliance of patients with chronic diseases, and limited drug information for healthcare providers.

### The “define” phase

**Fig 3:**
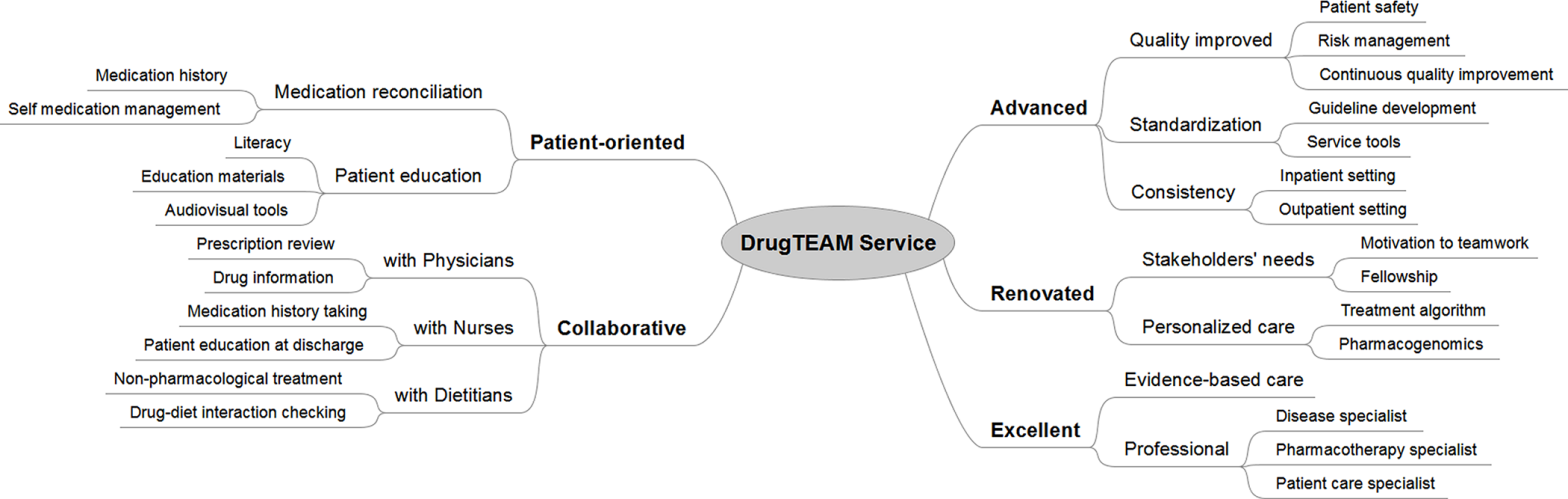
Service concepts based on mind mapping analysis. (Abbr.: DrugTEAM, drug therapy evaluation and management).

To address these problems, we derived five core concepts, which were used to name our new approach to MTC services, called Patient-oriented, Collaborative, Advanced, Renovated, and Excellent (P-CARE) service (Fig 3). The aim of our collaborative MTC service model is to provide longitudinal care for chronic disease patients who have a high risk of drug-related problems. Additionally, we created a brand name for this service to reflect the purpose and characteristics of our service model, called Drug Therapy and Management, which is abbreviated to “DrugTEAM”.

### The “design” phase

We designed four patient-centered services, as described briefly in Table 1. These services include: 1) a medication reconciliation (MR) service to reduce prescription discrepancies, 2) a medication evaluation and management (MEM) service to promote appropriate use of medications, 3) an evidence-based drug information (EB-DI) service to provide clinical support for patient care, and 4) a pharmaceutical care transition (PCT) service to improve patient compliance. The main provider of the service is a professional pharmacist, and the other healthcare professionals and the patients are colleagues and consumers.

**Table 1:** Service types and tasks in our collaborative multidisciplinary DrugTEAM service model.

Services	Service Tasks
Medication Reconciliation (MR) service	[Task 1] Collect the patient’s medication profile, which is a list of medications the patient has previously taken [Task 2] Check the duplication of treatment and any patient history of drug allergies or adverse reactions to drugs [Task 3] Communicate with the physicians, when there are discrepancies with the medications that are prescribed after admission [Task 4] Document the MR service results
Medication Evaluation and Management (MEM) service	[Task 1] Find drug related problems after reviewing the prescribed medications [Task 2] Assess the appropriateness of the treatments [Task 3] Recommend a plan of appropriate drug therapies based on the evidence [Task 4] Monitor the patient’s clinical changes whether the recommendations are reflected in the prescription lists [Task 5] Document the MEM service results
Evidence-based Drug Information (EB-DI) service	[Task 1] Document evidence-based drug information during pharmacotherapy [Task 2] Intervene rapidly to a physician’s request for patient-specific drug information
Pharmaceutical Care Transition (PCT) service	[Task 1] Collect all of a patient’s medications [Task 2] Check the duplication of treatment and compare that list with the admission and/or pre-admission prescriptions [Task 3] Communicate with physicians about discrepancies between prescriptions of pre- and after discharge [Task 4] Counsel to patients for improving health knowledge and medication compliance [Task 5] Document the PCT service results

Abbr.: DrugTEAM, drug therapy evaluation and management

### The “develop” phase

**Fig 4:**
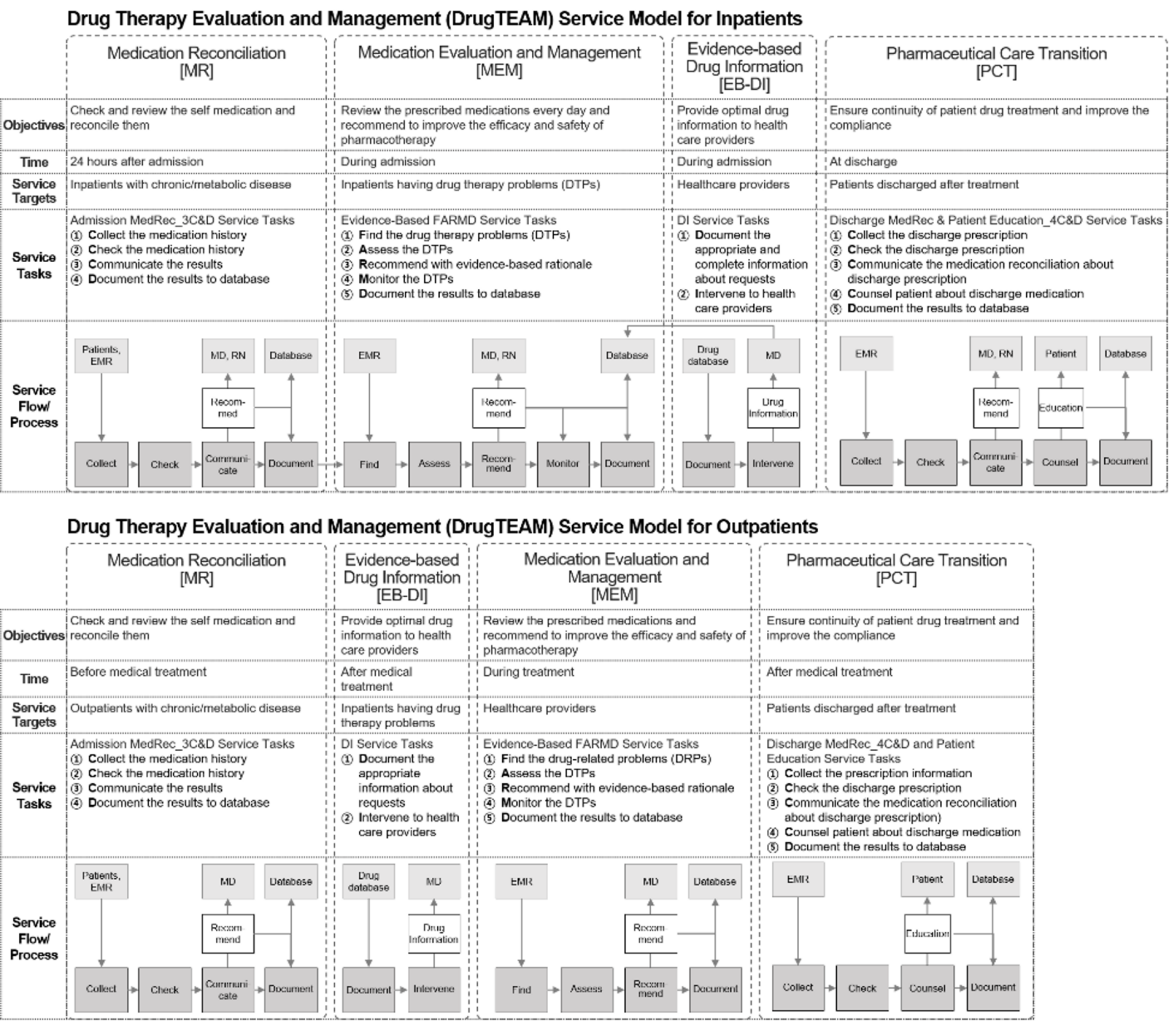
A collaborative multidisciplinary DrugTEAM service models for inpatients and outpatients. (Abbr.: EMR, electronic medical record; MD, medical doctor; RN, registered nurse).

The four major services (MR, MEM, EB-DI, and PCT) were implemented in order according to the patient’s time spent in hospital. The MR service begins at admission, followed by the MEM and EB-DI services during hospitalization. Finally, the discharge-PCT service is provided upon discharge. After discharge, when a patient returns to an ambulatory care clinic for a routine follow-up, a new set of services is started. Therefore, our service model can be applied to both inpatient and outpatient settings to provide longitudinal and continuous care. The final model is presented in blueprints, with consideration for the timing and locations of service requirements of each hospital setting (Fig 4(A) and 4(B) for inpatient and outpatient services, respectively).

#### Medication reconciliation service

The objective of the MR service is to accurately and completely resolve potential problems by ensuring that all medications are correct and preventing unintended changes or omissions of medications. The service involves the “3C&D” tasks, which are to Collect, Check, Communicate, and Document a patient’s medication history. When a patient is first admitted to the hospital, a pharmacist will collect the patient’s medication profile and makes a list of medications, including prescription medications, over-the-counter drugs, vitamins, and herbal medications. The pharmacist will then check for omissions, duplications, and dosing errors and reconcile any discrepancies between the medications that are prescribed before and after admission. If discrepancies are found, the pharmacist will communicate with the appropriate physicians and then documents the reasons for treatment changes. All tasks are performed within 24 hours of a patient’s admission to the hospital.

#### Medication evaluation and management service

The objective of the MEM service is to provide safe and effective treatments through collaboration with a team of healthcare providers. The service consists of defined tasks, specifically Find, Assess, Recommend, Monitor, and Document (FARM-D) the medications prescribed to a patient. A pharmacist finds and assesses the appropriateness of prescribed medications by considering any changes in the clinical status of a patient, using the Medication Appropriateness Index [26, 27]. Based on gathered evidence, the pharmacist then communicates and recommends a plan for solving drug-related problems to the team members. After that, the physician and pharmacist monitor whether the recommendations are reflected in the prescription within a 24-hour time period. All service tasks are documented and information about the treatment plan is provided to other members of the multidisciplinary team.

#### Evidence-based drug information service

The EB-DI service provides timely, evidence-based, and up-to-date information to healthcare providers to promote rational medication use. If there are any questions about drug information during treatment, a clinical pharmacist should immediately search tertiary, secondary, then primary resources, to find information that is clinically relevant to the patient-specific problem. Drug information may include the alternative treatments, appropriate dosing and administration, drug toxicity (i.e., adverse effects), drug-drug and drug-food interactions, dosing adjustments for renal or hepatic insufficiencies, drug use during pregnancy and lactation, pharmacokinetics, drug monitoring for efficacy and toxicity, and intravenous drug compatibility. After gathering sufficient evidence and performing critical review, the pharmacist responds to the request as soon as possible.

#### Pharmaceutical care transition service

The PCT service includes five tasks that aim to facilitate the transition to continuous treatment and improve compliance with medications after discharge. The tasks are abbreviated as 4C&D, and the first 3Cs and D are the same as in the MR service: Collect, Check, Communicate, and Document. When care is transferred, pharmacist reconcile the prescribed medication at discharge with the patient had been taking before admission. Then, the final “C” task is to provide counseling to patients and their caregiver, to improve medication knowledge and compliance. Patient counseling may include instructions on specific ways to take medications and skills to self-manage their disease. All educational materials should be written simply and clearly to facilitate patient understanding. To increase patient compliance, pill boxes or medication diary can be made available. Finally, after completion of all tasks, the counseling contents and service results should be documented.

## Discussion

In this study, we used a “4D” service design framework and various service design tools to visualize a collaborative MTC service model. Our essential goal in the service design was to gain stakeholder insights about unmet needs and to create innovative services based on those needs. Using the service design process, we discovered the unmet needs in current service models, defined service concepts and objectives, designed service prototypes, and developed an employable service model. This study applies a service design framework to the development of a customer-oriented and collaborative MTC service model for medication management in chronic disease patients.

In our final service model, our collaborative team was made up of multidisciplinary professional healthcare providers. The service provider is a professional pharmacist, the target and co-providers of the service are a doctor and nurse, and the ultimate beneficiary of the service is the patient. Based on our desk and field research, we found that stakeholders want pharmacists to provide patient-individualized services to minimize the risk of drug-related problems and improve medication compliance. Lauffenburger *et al*. suggested that clinical pharmacists play a key role in preventing drug-related adverse events for patients and that physicians want to build collaborative relationships with pharmacists to strengthen professional communication [28]. In particular, collaborative MTC services are in high demand for patients treated with polypharmacy, because polypharmacy is associated with an increased risk of prescription of potentially inappropriate medications, medication duplication, and drug-drug interactions [29]. Thus, our service model was developed to reduce these medication-related problems, improve medication compliance, and consequently maximize the safety and efficacy of pharmacotherapy. Finally, we proposed four services to satisfy the unmet needs of stakeholders that could be applied to both inpatient and outpatient settings. Furthermore, we intend to develop a longitudinal service model that can deliver timely and continuous care in a cycle system.

As the first step in the service model, the MR service is essential to resolve prescription discrepancies and ensure appropriate medication use during hospitalization. In our study, the physicians and nurses had difficulties with recording patient medication histories, which can lead to prescription discrepancies. The Joint Commission has recommended that the best MR service has a complete understanding and accurate record of the patient’s medication history [30]. A recent meta-analysis suggested that pharmacist-oriented MR services were effective in reducing hospital revisits and readmissions [31], as well as saving estimated annual costs and hospital expenses [32]. Thus, pharmacist-mediated prescription reconciliation can decrease medication errors, reduce hospital stays, and lead to healthcare cost savings [33]. The second step, the MEM service, is one of the most important tasks for administering good patient care. Because most inpatients have complex pharmacotherapy regimens and rapid changes in their conditions, they require special care from a qualified collaborative team. The MEM service identifies any potentially inappropriate medication uses and improves medication management by using evidence-based practices, through daily monitoring of the patient’s clinical status. It is well-known that medication therapy management (MTM) programs have helped identify and resolve numerous drug therapy problems, resulting in medical cost savings and reduced hospital stays [4, 6, 34, 35]. In addition to the MEM service, it is important that the timely provision of pharmacist evidence-based information focused on patient-specific problems decreases the risk of medication errors [36]. Finally, the other core role of the pharmacist is to provide PCT service which connects patient care when care shifts from one setting to another, such as from a hospital to the home, ambulatory clinic, or other healthcare facility. Furthermore, a pharmacist educates patients having greater medication knowledge and encourages medication compliance, which leads to improved long-term treatment outcomes [37, 38].

Service design is a powerful tool, because it allows stakeholders to directly address their needs by participating in model development [12]. The use of this methodology has been supported by many other fields, including business marketing, public health, and finance. This methodology has improved productivity, saved energy, and reduced costs for end users, resulting in more efficient and appealing services [39]. Nevertheless, it is necessary to consider the specific needs of the clinical environment when applying our service model. Additionally, applying this service to the clinical environment may increase the work of pharmacists and incur additional costs for the patient. To provide professional and standardized service, a pharmacist must have expertise in the disease through more than a certain amount of time. Several randomized controlled trials, conducted with a MTC service model, have shown that total costs (both direct medical costs and indirect costs) for each patient in the service user group were higher than costs for a control group [40–43]. However, because the service group had statistically significant improvements in humanistic and/or clinical outcomes with lower associated costs, pharmaceutical care service was strongly favored. To reconcile the remaining concerns, we are planning a large number of clinical studies to assess whether this service model can improve patients’ clinical and humanistic outcomes, or save total costs to the healthcare system.

In conclusion, we used a service design framework to develop a MTC service model that can be applied in inpatient or outpatient settings, based on the needs of stakeholders. The service model can also be applied to the treatment of chronic diseases that have high a risk of drug-related problems and require comprehensive medication management. We expect our service to reduce the length of hospital stays and to improve the quality of life for patients by enabling comprehensive medication management. Furthermore, we believe this patient-centered service model can improve patient outcomes by enhancing the professional relationships between pharmacists and other healthcare providers.

## References

[1] Zhang C, Zhang L, Huang L, Luo R, Wen J. Clinical pharmacists on medical care of pediatric inpatients: a single-center randomized controlled trial. PloS one. 2012;7(1):e30856 10.1371/journal.pone.0030856 22292061 PMC3264625

[2] Wiedenmayer K, Summers SR, Mackie AC, Gous GSA, Everard M, Tromp D. Pharmacists in the health care team: a policy perspective. Handbook of developing pharmacy practice: a focus on patient care Department of Medicines Policy and Standards, Geneva, Switzerland: World Health Organization; 2006 pp. 7–8.

[3] WHO. The role of the pharmacist in the health care system. New Delhi, India: World Health Organization, 1998 December 13–16, 1988. Report No.

[4] Isetts BJ, Schondelmeyer SW, Artz MB, Lenarz LA, Heaton AH, Wadd WB, et al Clinical and economic outcomes of medication therapy management services: the Minnesota experience. J Am Pharm Assoc. 2008;48(2):203–211. 10.1331/JAPhA.2008.07108 18359733

[5] Moore JM, Shartle D, Faudskar L, Matlin OS, Brennan TA. Impact of a patient-centered pharmacy program and intervention in a high-risk group. J Manag Care Pharm. 2013;19(3):228–236. doi: 10.18553/jmcp.2013.19.3.228 23537457 10.18553/jmcp.2013.19.3.228 PMC10438107

[6] Reinke T. Medication therapy management program in N.C. saves $13 million. Managed care. 2011;20(10):17–18. 22111478

[7] Bauters T. The role of the pharmacist in a multidisciplinary team. Hospital Pharmacy Europe. 2014 5 6, 2014.

[8] Epstein NE. Multidisciplinary in-hospital teams improve patient outcomes: A review. Surg Neurol Int. 2014;5(Suppl 7):S295–303. 10.4103/2152-7806.139612 25289149 PMC4173201

[9] Ndoro S. Effective multidisciplinary working: the key to high-quality care. Br J Nurs. 2014;23(13):724–727. doi: 10.12968/bjon.2014.23.13.724 25072333 10.12968/bjon.2014.23.13.724

[10] Lee IH, Rhie SJ, Je NK, Rhew KY, Ji E, Oh JM, et al Perceived needs of pharmaceutical care services among healthcare professionals in South Korea: a qualitative study. Int J Clin Pharm. 2016;38(5):1219–1229. 10.1007/s11096-016-0355-9 27581712

[11] Karine Freire DS. Service design and healthcare innovation: from consumption, to co-production to co-creation. Linkoping, Sweden: Nordic Service Design Conference; 2010. p. 1–11.

[12] Griffiths J, Maggs H, George E. Stakeholder Involvement. Switzerland: World Health Organization; 2008 p. 1–41.

[13] Tan EC, Stewart K, Elliott RA, George J. Stakeholder experiences with general practice pharmacist services: a qualitative study. BMJ open. 2013;3(9):e003214 10.1136/bmjopen-2013-003214 24030867 PMC3773653

[14] Rhew K, Han N, Cho KT, Yoon JH, Oh JM. Prioritization of diseases for the development of a pharmaceutical care service model in South Korea using the analytic hierarchy process. Int J Clin Pharmacol Ther. 2017;55(11):866–874. 10.5414/CP202964 28853698

[15] Polaine A. Chapter 4: Turning Research into Insight and Action Service design: from insight to implementation. Brooklyn, NY: Rosenfeld Media; 2013 pp. 58–60.

[16] Reason B, Løvlie L, Flu MB. Chpater 3. The Customer Story: Understanding Customers Better Provides the Basis for Customer-Driven Service Improvement and Innovation Service design for business: a practical guide to optimizing the customer experience. Hoboken, N.J.: Wiley; 2016 pp. 58–86.

[17] Kang J, Rhew K, Oh JM, Han N, Lee IH, Je NK, et al Satisfaction and expressed needs of pharmaceutical care services and challenges recognized by patients in South Korea. Patient Prefer Adherence. 2017;11:1381–1388. 10.2147/PPA.S141562 28860721 PMC5565375

[18] Jane R, Liz S. Qualitative data analysis for applied policy research In: Bryman A BR, editor. The Qualitative Researcher's Companion. London: SAGE Publications, Inc; 2002 pp. 305–330.

[19] National Centre for Social Research. Framework: the qualitative data analysis tool 2010. Available from: http://www.natcen.ac.uk/our-expertise/methods-expertise/qualitative/framework.

[20] Guest G, MacQueen, Namey. Introduction to Applied Thematic Analysis Applied Thematic Analysis. London: SAGE Publications, Inc; 2012 pp. 3–20.

[21] American Society of Health-System Pharmacists. ASHP Statement on Pharmaceutical Care. ASHP Statements and Guidelines [Internet]. 2014:[266–268 pp.]. Available from: http://www.ashp.org/doclibrary/bestpractices/orgstpharmcare.aspx.

[22] American Society of Hospital Pharmacists. Draft statement on pharmaceutical care. Am J Hosp Pharm. 1993;50(1):126–128. 8427270

[23] Inker LA, Astor BC, Fox CH, Isakova T, Lash JP, Peralta CA, et al KDOQI US commentary on the 2012 KDIGO clinical practice guideline for the evaluation and management of CKD. Am J Kidney Dis. 2014;63(5):713–735. 10.1053/j.ajkd.2014.01.416 24647050

[24] Chamberlain JJ, Herman WH, Leal S, Rhinehart AS, Shubrook JH, Skolnik N, et al Pharmacologic Therapy for Type 2 Diabetes: Synopsis of the 2017 American Diabetes Association Standards of Medical Care in Diabetes. Ann Intern Med. 2017;166(8):572–578. 10.7326/M16-2937 28288484

[25] Kovell LC, Ahmed HM, Misra S, Whelton SP, Prokopowicz GP, Blumenthal RS, et al US Hypertension Management Guidelines: A Review of the Recent Past and Recommendations for the Future. J Am Heart Assoc. 2015;4(12). pii: e002315 10.1161/JAHA.115.002315 26643500 PMC4845275

[26] Samsa GP, Hanlon JT, Schmader KE, Weinberger M, Clipp EC, Uttech KM, et al A summated score for the medication appropriateness index: development and assessment of clinimetric properties including content validity. J Clin Epidemiol. 1994;47(8):891–896. 10.1016/0895-4356(94)90192-9 7730892

[27] Hanlon JT, Schmader KE, Samsa GP, Weinberger M, Uttech KM, Lewis IK, et al A method for assessing drug therapy appropriateness. J Clin Epidemiol. 1992;45(10):1045–1051. 10.1016/0895-4356(92)90144-C 1474400

[28] Lauffenburger JC, Vu MB, Burkhart JI, Weinberger M, Roth MT. Design of a medication therapy management program for Medicare beneficiaries: qualitative findings from patients and physicians. Am J Geriatr Pharmacother. 2012;10(2):129–138. 10.1016/j.amjopharm.2012.01.002 22284582 PMC3322273

[29] Sehgal V, Bajwa SJ, Sehgal R, Bajaj A, Khaira U, Kresse V. Polypharmacy and potentially inappropriate medication use as the precipitating factor in readmissions to the hospital. J Family Med Prim Care. 2013;2(2):194–199. 10.4103/2249-4863.117423 24479078 PMC3894035

[30] Dolin RH, Giannone G, Schadow G. Enabling joint commission medication reconciliation objectives with the HL7 / ASTM Continuity of Care Document standard. AMIA Annu Symp Proc. 2007:186–190. 18693823 PMC2813669

[31] Mekonnen AB, McLachlan AJ, Brien JA. Effectiveness of pharmacist-led medication reconciliation programmes on clinical outcomes at hospital transitions: a systematic review and meta-analysis. BMJ open. 2016;6(2):e010003 10.1136/bmjopen-2015-010003 26908524 PMC4769405

[32] Smith M, Giuliano MR, Starkowski MP. In Connecticut: improving patient medication management in primary care. Health affairs. 2011;30(4):646–654. 10.1377/hlthaff.2011.0002 21471485

[33] Bond CA, Raehl CL, Franke T. Medication errors in United States hospitals. Pharmacotherapy. 2001;21(9):1023–1036. 10.1592/phco.21.13.1023.34617 11560192

[34] Ross LA, Bloodworth LS. Patient-centered health care using pharmacist-delivered medication therapy management in rural Mississippi. J Am Pharm Assoc (2003). 2012;52(6):802–809. 10.1331/JAPhA.2012.10192 23229968

[35] Wolf C, Pauly A, Mayr A, Gromer T, Lenz B, Kornhuber J, et al Pharmacist-Led Medication Reviews to Identify and Collaboratively Resolve Drug-Related Problems in Psychiatry—A Controlled, Clinical Trial. PloS one. 2015;10(11):e0142011 10.1371/journal.pone.0142011 26544202 PMC4636233

[36] Cardoni AA, Thompson TJ. Impact of drug information services on patient care. Am J Hosp Pharm. 1978;35(10):1233–1237. 696731

[37] Osterberg L, Blaschke T. Adherence to medication. N Engl J Med. 2005;353(5):487–497. 10.1056/NEJMra050100 16079372

[38] Lee JK, Grace KA, Taylor AJ. Effect of a pharmacy care program on medication adherence and persistence, blood pressure, and low-density lipoprotein cholesterol: a randomized controlled trial. JAMA. 2006;296(21):2563–2571. 10.1001/jama.296.21.joc60162 17101639

[39] Enninga T, Manschot M, Gessel Cv, Gijbels J, Lugt Rvd, Visser FS, et al Service Design: insights from nine case studies: HU University of Applied Sciences Utrecht; 2013 Available from: https://www.stby.eu/wp_15/wp-content/uploads/2013/12/Service-Design-insights-from-nine-case-studies.pdf.

[40] Elliott RA, Barber N, Clifford S, Horne R, Hartley E. The cost effectiveness of a telephone-based pharmacy advisory service to improve adherence to newly prescribed medicines. Pharm World Sci. 2008;30(1):17–23. 10.1007/s11096-007-9134-y 17557211

[41] Jódar-Sánchez F, Malet-Larrea A, Martín JJ, García-Mochón L, López Del Amo MP, Martínez-Martínez F, et al Cost-utility analysis of a medication review with follow-up service for older adults with polypharmacy in community pharmacies in Spain: the conSIGUE program. Pharmacoeconomics. 2015;33(6):599–610. 10.1007/s40273-015-0270-2 25774017

[42] McLean W, Gillis J, Waller R. The BC Community Pharmacy Asthma Study: A study of clinical, economic and holistic outcomes influenced by an asthma care protocol provided by specially trained community pharmacists in British Columbia. Can Respir J. 2003;10(4):195–202. 10.1155/2003/736042 12851665

[43] van Boven JF, Tommelein E, Boussery K, Mehuys E, Vegter S, Brusselle GG, et al Improving inhaler adherence in patients with chronic obstructive pulmonary disease: a cost-effectiveness analysis. Respir Res. 2014;15:66 10.1186/1465-9921-15-66 24929799 PMC4067522

